# Low-Level Elevations of Procalcitonin Are Associated with Increased Mortality in Acute Heart Failure Patients, Independent of Concomitant Infection

**DOI:** 10.3390/life11121429

**Published:** 2021-12-18

**Authors:** Fabrice F. Darche, Moritz Biener, Matthias Müller-Hennessen, Rasmus Rivinius, Kiril M. Stoyanov, Barbara R. Milles, Hugo A. Katus, Norbert Frey, Evangelos Giannitsis

**Affiliations:** 1 Department of Cardiology, Angiology and Pneumology, Heidelberg University Hospital, 69120 Heidelberg, Germany; Moritz.Biener@med.uni-heidelberg.de (M.B.); Matthias.Mueller-Hennessen@med.uni-heidelberg.de (M.M.-H.); rasmus.rivinius@med.uni-heidelberg.de (R.R.); Kiril.Stoyanov@med.uni-heidelberg.de (K.M.S.); BarbaraRuth.Milles@med.uni-heidelberg.de (B.R.M.); Hugo.Katus@med.uni-heidelberg.de (H.A.K.); Norbert.Frey@med.uni-heidelberg.de (N.F.); evangelos.giannitsis@med.uni-heidelberg.de (E.G.); 2 German Center for Cardiovascular Research (DZHK), Partner Site Heidelberg/Mannheim, 69120 Heidelberg, Germany

**Keywords:** AHF, PCT, NT-proBNP, all-cause mortality

## Abstract

We aimed to evaluate the prognostic value of procalcitonin (PCT) in acute heart failure (AHF) patients, especially in those without underlying infection. We enrolled patients presenting with acute dyspnea to the emergency department (ED) of Heidelberg University Hospital and studied the prognostic role of PCT on all-cause death. Of 312 patients, AHF was diagnosed in 139 patients. Of these, 125 patients had AHF without signs of infection, and 14 had AHF complicated by respiratory or other infection. The optimal prognostic PCT cutoff value for mortality prediction was calculated by a receiver operating characteristics curve. In patients with AHF, the prognostic PCT cutoff value was 0.08 ng/mL. The Kaplan–Meier survival analysis showed that AHF patients with PCT values > 0.08 ng/mL had a higher all-cause mortality at 120 days than those with PCT values ≤ 0.08 ng/mL (log-rank *p* = 0.0123). Similar results could be obtained after subdivision into AHF patients with and without signs of overt infection. In both cases, mortality was higher in patients with PCT levels above the prognostic PCT cutoff than in those with values ranging below this threshold. Moreover, we show that the prognostic PCT cutoff values for mortality prediction ranged below the established PCT cutoff for the guidance of antibiotic therapy. In conclusion, the data of our study revealed that low-level elevations of PCT were associated with an increased mortality in patients with AHF, irrespective of concomitant respiratory or other infection. PCT should thus be further used as a marker in the risk stratification of AHF.

## 1. Introduction

The rapid and accurate diagnosis of acute heart failure (AHF) and discrimination from respiratory infection is paramount in patients presenting with acute dyspnea at the emergency department (ED) in order to initiate effective and early treatment for heart failure, respiratory infection, or both. Considering that acute dyspnea is not specific for AHF, the elucidation of the underlying causes for acute dyspnea remains a challenge. A delay of time has a negative impact on patients’ clinical outcome [1]. Natriuretic peptides (NPs), such as brain-type natriuretic peptide (BNP), N-terminal pro brain-type natriuretic peptide (NT-proBNP), or atrial natriuretic peptide (ANP), play an important role in the diagnostic work-up and identification of AHF within dyspneic patients, and have been consequently recommended by practice guidelines [2,3,4,5]. In a previous prospective study, which had enrolled patients with acute dyspnea, we showed that the mid-regional pro-atrial natriuretic peptide (MR-proANP) may be used as an equitable alternative to NT-proBNP for the diagnosis of AHF [6]. Interestingly, the diagnostic performance of both NT-proBNP and MR-proANP was not affected by comorbidities like older age, renal dysfunction, obesity, atrial fibrillation, and paced rhythm [6]. Moreover, we demonstrated that both biomarkers were capable of predicting mortality in AHF patients [6]. Apart from NPs, soluble ST2 and cardiac troponins confer supplemental prognostic information in patients with AHF [7]. Several previous trials demonstrated that PCT improved the discrimination of respiratory infection in patients with dyspnea or confirmed acute heart failure when levels were close to the recommended thresholds that signal relevant bacteremia [8,9,10]. The prognostic value of procalcitonin (PCT) in AHF patients without overt signs of respiratory infection is still unresolved [8]. Recently, the hypothesis was raised that low-level PCT elevations may be encountered in AHF without respiratory infection [11,12]. The prognostic role of these low-level elevations is, however, unsettled. In this single-center prospective study, we therefore studied the prognostic performance of PCT to predict all-cause mortality in AHF patients, irrespective of concomitant infection.

## 2. Materials and Methods

From May 2013 to November 2014, we enrolled 312 patients presenting to the ED of Heidelberg University with an acute onset of dyspnea or an acute deterioration of pre-existing dyspnea within the previous 14 days before admission [6]. Patients’ diagnostic work-up consisted of medical history, physical examination, and laboratory testing including PCT, NT-proBNP, ECG, and chest radiography [6]. Additional diagnostic exams were left at the discretion of the attending emergency physician [6]. Patients were followed up for a period of 120 days.

### 2.1. Adjudication of Final Diagnoses

The adjudication of final diagnoses was performed by two cardiologists as previously described [6]. In brief, they independently reviewed all medical records and independently adjudicated a final diagnosis to each patient of the study cohort by referring to data on chest radiography, lung computed tomography and/or multi-slice-CT angiography, 2-D echocardiography, abdominal ultrasonography, and cardiac catheterization [6]. AHF patients were classified as heart failure with preserved ejection fraction (HFpEF) if systolic left ventricular (LV) function was normal and as heart failure with reduced (HFrEF) or mildly reduced (HFmrEF) ejection fraction if systolic LV function was impaired [5]. Moreover, the ProBNP Investigation of Dyspnea in the Emergency Department (PRIDE) score was calculated retrospectively to provide an objective diagnosis of AHF [6,13]. For the confirmation of infectious diseases, data including C-reactive protein, leukocytes, temperature at presentation, location of infection, identification of pathogens from blood cultures, urine, or sputum, imaging information from chest radiography or chest CT, abdominal ultrasound, urine sediment and urine culture, and initiation and cessation of antimicrobial therapies were studied and interpreted [6].

The study was observational and all medical decisions, therapies, or further diagnostic work-up were left to the discretion of the attending emergency physician [6]. It was performed according to the principles of the Declaration of Helsinki and approved by the local ethics committee (Heidelberg, registration number S-117/2013). Written informed consent was obtained from all participating patients [6].

### 2.2. Biomarker Testing

NT-proBNP was measured by the Stratus^®^ CS Acute Care™ NT-proBNP assay (Siemens AG, Berlin and Munich, Germany) based on the sandwich chemiluminescence technique, which is described in detail elsewhere [14,15] and which has been used in other studies [14,16,17,18]. In addition, PCT was determined from the admission blood sample in all patients routinely, and results were available for interpretation by the attending physician during ED stay. A PCT cutoff of 0.25 ng/mL was applied to detect severe bacterial infection and to initiate antimicrobial therapy [9]. C-reactive protein (CRP) and white blood cells (WBC) were determined to rule out infections.

### 2.3. Diagnostic Performance of NT-ProBNP

We evaluated the diagnostic performance of NT-proBNP at the recommended cutoff value for AHF rule-in [4,19]. In addition, the diagnostic performance of NT-proBNP was evaluated by a receiver operating characteristic (ROC) curve, and the ROC optimal cutoff value was calculated, as published elsewhere [6].

### 2.4. Prognostic Value of NT-ProBNP in the Overall Study Population

The ROC optimal cutoff value for NT-proBNP was used to dichotomize outcomes using Kaplan–Meier survival analysis.

### 2.5. PCT for the Discrimination of Infection

We applied the recommended cutoff of 0.25 ng/mL [9] to test the diagnostic performance of PCT in the overall study population and in AHF patients. We calculated ROC statistics as well as sensitivities, specificities, and positive and negative predictive values at the pre-specified cutoff. Moreover, we calculated the ROC optimal PCT cutoffs for the diagnosis of infections in the overall study population and in AHF patients.

### 2.6. Diagnostic Added Value of NT-ProBNP and PCT

To evaluate the diagnostic added value of NT-proBNP and PCT, we compared ROC curves with the logistic regression of NT-proBNP, PCT, and NT-proBNP + PCT. To enable comparison, an ROC curve analysis was performed in patients with AHF and respiratory or other infection.

### 2.7. Prognostic Value of PCT in the Entire Study Cohort and in AHF with or without Established Respiratory or Other Infection

We calculated the optimal prognostic cutoff of PCT in the entire study cohort and in AHF using ROC statistics. Based on the ROC optimal cutoff, all-cause mortality between PCT positive and PCT negative cohorts was calculated by log-rank using Kaplan–Meier survival curves. Moreover, we evaluated the prognostic value of PCT in both AHF subgroups with and without established respiratory or other infection by ROC and Kaplan–Meier survival curves as well as by interaction testing using probit regression.

### 2.8. Assessment of the Independent Prognostic Performance of NT-ProBNP and PCT

We performed Cox regression analysis to assess the independent prognostic value of NT-proBNP and PCT regarding mortality in the entire study cohort. NT-proBNP positive patients were defined as patients meeting the age-dependent rule-in criteria for AHF. PCT positive patients were patients with PCT values ranging above the ROC optimal PCT cutoff value for mortality prediction within the whole study population.

### 2.9. Statistical Analysis

The pairwise comparison of categorical or continuous variables was performed using an χ^2^ test or a D’Agostino–Pearson test, respectively. Continuous variables are presented as arithmetic mean ± standard error of the mean (SEM). We determined the diagnostic performance for the diagnosis of infections from the ROC curve on the basis of continuously measured PCT levels using the test of DeLong et al. [20]. The ROC optimal cutoff value was calculated using the point closest to the upper left corner according to the method proposed by Zweig et al. [21]. In addition, we calculated sensitivities, specificities, and negative and positive predictive values for the diagnosis of infections or AHF. ROC curves with logistic regression were performed to evaluate the diagnostic added value of NT-proBNP or PCT. Kaplan–Meier curves were calculated for the estimation of survival. Cox regression was performed to analyze if NT-proBNP and PCT influenced survival independently of each other. Probit regression was used for the interaction testing of PCT and mortality probability. The MedCalc 11.1 (MedCalc software, Mariakerke, Belgium) statistical software package was used. A *p*-value < 0.05 was considered statistically significant.

## 3. Results

### 3.1. Characteristics of AHF Patients with and without Respiratory or Other Infection

We enrolled 312 patients presenting with acute dyspnea to the ED. Within the study population, the adjudicated diagnosis was AHF in 139 patients (44.6%). Concomitant respiratory infections were present in 14 AHF patients (4.5% of the study population or 10.1% of all AHF patients), whereas the majority (*n* = 125, 40.1% of the study population or 89.9% of all AHF patients) did not have any respiratory or any other relevant bacterial infection (uncomplicated AHF patients). Dyspneic patients in whom AHF was excluded had isolated respiratory infections comprising pneumonia (*n* = 25), asthma (*n* = 31), acute coronary syndrome (*n* = 33) (STEMI, *n* = 1, NSTEMI, *n* = 4, unstable angina, *n* = 28), pulmonary embolism (*n* = 22), arrhythmias (*n* = 21), and remaining diagnoses including structural and congenital heart diseases, hypertensive crisis, malignancies, rheumatological, hematological and auto-immune diseases, atypical chest pain, trauma, and neurological and psychosomatic diseases (*n* = 41), as previously described [6]. Table 1 compares patients’ characteristics between groups with uncomplicated AHF (*n* = 125) and groups with AHF and respiratory or other infection (*n* = 14). There were no statistically significant differences regarding gender, advanced age, kidney function, or the cardiovascular risk factors arterial hypertension, dyslipidemia, diabetes mellitus, nicotine consumption, and obesity between the two groups (Table 1). Moreover, both groups comprised a similar high amount of patients with reduced systolic LV function (77.7% of patients with uncomplicated AHF and 76.9% of AHF patients with respiratory or other infection, *p* = 0.9502, Table 1), classified as HFrEF or HFmrEF [5]. The remaining patients in both AHF groups had HFpEF [5]. In comparison, only 33.1% of dyspneic patients with diagnoses other than AHF had impaired LV function [6]. As expected, NT-proBNP values were significantly higher in patients with a diagnosis of AHF compared to those where AHF was excluded (9.898,3 ± 1.077,7 ng/L vs. 1.445,3 ± 257.1 ng/L, *p* < 0.001). Interestingly, AHF patients with respiratory or other infection had higher NT-proBNP values than uncomplicated AHF patients (8.307,4 ± 819.0 ng/L vs. 24.102,6 ± 6.930,8 ng/L, *p* < 0.001) (Table 1). Likewise, PCT values were significantly higher among patients with established respiratory or other infection compared to those where infection was ruled out (0.84 ± 0.45 ng/mL vs. 0.09 ± 0.01 ng/mL, *p* < 0.001). Similar results could be raised in the AHF subgroup, where PCT values were significantly higher in AHF patients with respiratory or other infection than in those without any infection (0.39 ± 0.23 ng/mL vs. 0.10 ± 0.03 ng/mL, *p* < 0.001) (Table 1). Importantly, AHF patients without any infection showed normal CRP and WBC values compared to AHF patients with respiratory or other infection, where they were significantly elevated (Table 1).

### 3.2. Diagnostic Performance of NT-ProBNP at the Established Cutoff Values

**Figure 1:**
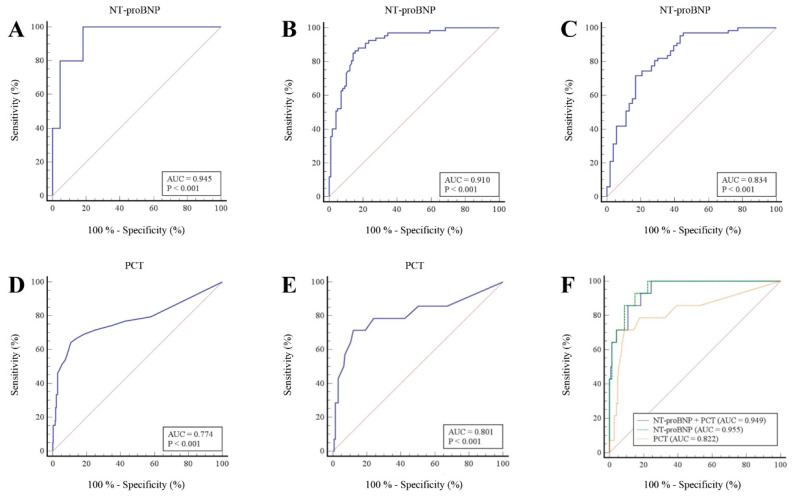
Diagnostic performances of NT-proBNP and PCT: diagnostic performances were evaluated by receiver operating characteristics curve (ROC). (**A**) Diagnostic performance of NT-proBNP to detect AHF in patients < 50 years of age. (**B**) Diagnostic performance of NT-proBNP to detect AHF in patients aged between 50 and 75 years. (**C**) Diagnostic performance of NT-proBNP to detect AHF in patients > 75 years of age. (**D**) Diagnostic performance of PCT to detect respiratory infections in the overall study population. (**E**) Diagnostic performance of PCT to detect respiratory infections in AHF patients. (**F**) Comparison of ROC curves with logistic regression; blue: diagnostic performance of NT-proBNP + PCT to detect AHF with respiratory or other infection; green: diagnostic performance of NT-proBNP to detect AHF with respiratory or other infection; orange: diagnostic performance of PCT to detect AHF with respiratory or other infection. AUC = area under the curve, NT-proBNP = N-terminal pro b-type natriuretic peptide, PCT = procalcitonin, AHF = acute heart failure, ROC = receiver operating characteristic.

The age-dependent [4,19] performances of NT-proBNP for the diagnosis of AHF within the overall study population were evaluated by ROC curves (Figure 1A–C). The area under the curve (AUC) value for NT-proBNP was 0.945 (95% confidence interval (CI): 0.784–0.996) for patients aged < 50 years (Figure 1A), 0.910 (95% CI: 0.856–0.949) for patients aged between 50 and 75 years (Figure 1B), and 0.834 (95% CI: 0.755–0.896) for patients aged > 75 years (Figure 1C). Table 2 summarizes the diagnostic performances of NT-proBNP at the established age-dependent AHF rule-in cutoff values (450 ng/L for patients aged < 50 years, 900 ng/L for patients aged between 50 and 75 years, and 1800 ng/L for patients aged > 75 years).

### 3.3. Prognostic Value of NT-ProBNP in the Overall Study Population

**Figure 2:**
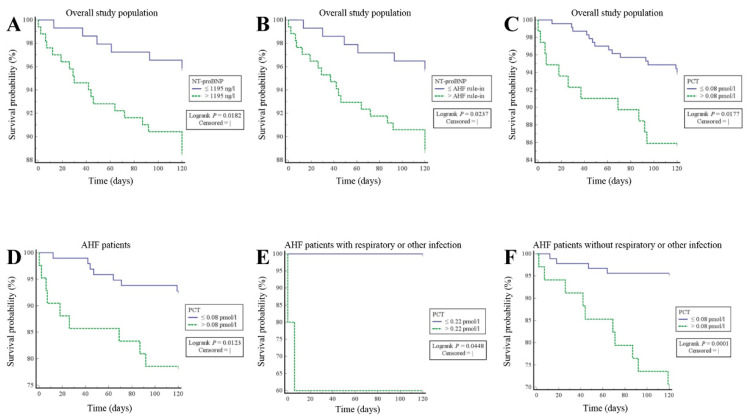
Mortality prediction by NT-proBNP and PCT: mortality assessment by Kaplan–Meier survival curve. (**A**,**B**) Mortality prediction by NT-proBNP in the entire study population (*n* = 312). Patients with NT-proBNP levels ranging above the ROC optimal (**A**) or the age-dependent AHF rule-in (**B**) cutoff have higher mortality than those with values below that threshold. (**C**–**F**) Mortality prediction by PCT in the entire study population (*n* = 312) (**C**), in AHF patients (*n* = 139) (**D**), in AHF patients with respiratory or other infection (*n* = 14) (**E**), as well as in AHF patients without any infection (*n* = 125) (**F**). In all groups, patients with PCT levels ranging above the prognostic ROC optimal cutoff have higher mortality than those with values below that threshold. NT-proBNP = N-terminal pro b-type natriuretic peptide, PCT = procalcitonin, AHF = acute heart failure, ROC = receiver operating characteristic.

Kaplan–Meier survival curves demonstrated a higher all-cause mortality at the ROC optimal cutoff [6] (log-rank *p* = 0.0182) (Figure 2A) and at the established age-dependent rule-in cutoff values [4,19] (log-rank *p* = 0.0237) (Figure 2B).

### 3.4. Diagnostic Performance of PCT for Respiratory or Other Infection in the Overall Study Population and in AHF Patients

The diagnostic performance of PCT for respiratory or other infection in the overall study population (Figure 1D) and in AHF patients (Figure 1E) was evaluated by ROC curves. The area under the curve (AUC) value for PCT was 0.774 (95% CI: 0.723–0.819) in the overall study population (Figure 1D) and 0.801 (95% CI: 0.725–0.864) in AHF patients (Figure 1E). We evaluated an ROC optimal threshold of 0.1 ng/mL in the overall study population and of 0.11 ng/mL in AHF patients. Moreover, we calculated the diagnostic performances of PCT at the usual 0.25 ng/mL cutoff established for the guidance of antibiotic therapy [9] and at the ROC optimal threshold (Figure 1D,E) in the overall study population and in AHF patients (Table 2).

### 3.5. Diagnostic Added Value of NT-ProBNP and PCT

The ROC curve analysis in patients with AHF and respiratory or other infection showed that the combination of NT-proBNP and PCT had a significantly higher diagnostic performance than PCT alone (AUC = 0.949 (95% CI: 0.903–0.977) for NT-proBNP + PCT vs. AUC = 0.822 (95% CI: 0.754–0.877) for PCT, *p* = 0.0440) (Figure 1F; Table 3). By contrast, there was no statistically significant difference between NT-proBNP + PCT and NT-proBNP regarding diagnostic performance (AUC = 0.949 (95% CI: 0.903–0.977) for NT-proBNP + PCT vs. AUC = 0.955 (95% CI: 0.911–0.981) for NT-proBNP, *p* = 0.1615) (Figure 1F; Table 3). Hence, NT-proBNP added diagnostic value to PCT (added value ΔAUC = 0.127) (Table 3).

### 3.6. Prognostic Value of PCT in the Entire Study Cohort and in AHF Patients with or without Respiratory or Other Infection

**Figure 3:**
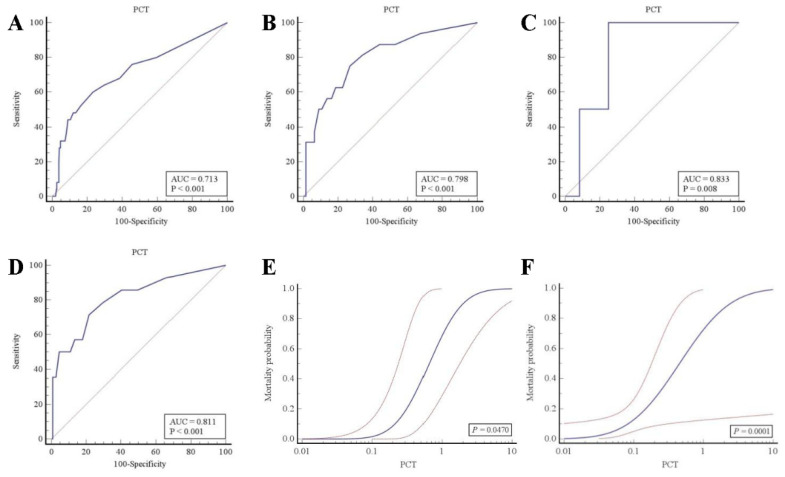
Prognostic performance of PCT by ROC curve and probit regression analysis: (**A**–**D**) prognostic performance of PCT by ROC curve analysis in the entire study population (**A**), in AHF patients (**B**), in AHF patients with respiratory or other infection (**C**), and in AHF patients without any infection (**D**). (**E**,**F**) Association between PCT concentration (ng/mL) and mortality in AHF patients with respiratory or other infection (**E**), and in AHF patients without any infection (**F**).

To assess the ability of PCT to predict mortality in patients with acute dyspnea, we calculated the prognostic performance of PCT in the entire study cohort and in AHF patients using ROC statistics. As shown by ROC curve analysis, the prognostic performance of PCT was excellent in the entire study cohort (AUC = 0.713 (95% CI: 0.660–0.763)) (Figure 3A) as well as in AHF patients (AUC = 0.798 (95% CI: 0.722–0.862)) (Figure 3B). Interestingly, the evaluated prognostic PCT cutoff was, at 0.08 ng/mL in both groups, relatively low, and hence far below the usual 0.25 ng/mL diagnostic PCT cutoff established for the guidance of antibiotic therapy [9]. Moreover, Kaplan–Meier survival curves showed that patients with PCT levels ranging above the relatively low prognostic ROC optimal cutoff of 0.08 ng/mL had a significantly higher mortality than those with PCT levels < 0.08 ng/mL in the entire study cohort (log-rank *p* = 0.0177) (Figure 2C) as well as in AHF patients (log-rank *p* = 0.0123) (Figure 2D). Furthermore, we wanted to evaluate if PCT was able to predict mortality in AHF patients without respiratory or other infection. Therefore, we compared the prognostic performance of PCT by ROC statistics in AHF patients with and without respiratory or other infection. In both groups, the prognostic performance of PCT was excellent, with an AUC of 0.833 (95% CI: 0.544–0.974) (Figure 3C) for AHF patients with respiratory or other infection and an AUC of 0.811 (95% CI: 0.731–0.875) (Figure 3D) for patients with uncomplicated AHF. The prognostic ROC optimal cutoff was 0.22 ng/mL for AHF patients with respiratory or other infection, whereas it was 0.08 ng/mL for patients with uncomplicated AHF. The Kaplan–Meier survival analysis indicated that mortality was increased in patients displaying PCT levels above the prognostic ROC optimal cutoff. These findings could be raised in AHF patients with respiratory or other infection (log-rank *p* = 0.0448) (Figure 2E) and, remarkably, also in patients without any infection at all (log-rank *p* = 0.0001) (Figure 2F). Finally, the probit regression analysis showed a significant rise of mortality probability upon PCT level increase in AHF patients with respiratory or other infection (*p* = 0.0470) (Figure 3E). Similar results could be obtained in uncomplicated AHF patients (*p* = 0.0001) (Figure 3F), with the difference being that the probit regression curve in uncomplicated AHF patients was shifted leftwards to lower PCT levels compared to that in AHF patients with respiratory or other infection.

### 3.7. Independent Prognostic Performance of NT-ProBNP and PCT in Patients with Acute Dyspnea

In the Cox regression analysis, PCT retained NT-proBNP positive patients with an increased mortality (*p* = 0.0205) (Table 4). Similarly, NT-proBNP selected PCT positive patients with an increased mortality (*p* = 0.0372) (Table 4). Hence, we could clearly demonstrate that PCT and NT-proBNP are able to predict, independently of each other, the mortality in patients with acute dyspnea.

## 4. Discussion

In this observational study of 312 dyspneic patients, we aimed to analyze whether PCT is associated with an increased all-cause mortality in patients with AHF, even if respiratory or any other relevant bacterial infection have been excluded. To rule out any respiratory or other infection in AHF patients at admission and during hospital stay, a comprehensive diagnostic work-up referring to clinical, laboratory, and instrument-based tools was applied. Importantly, the final adjudication of diagnosis was performed retrospectively by two cardiologists, who were not involved in patient management. In case of discordance, a third cardiologist was involved.

Our major findings are first the excellent diagnostic and prognostic performances of NT-proBNP at the established AHF rule-in cutoff values. Second, we found that the diagnostic performance of PCT to detect respiratory or other infection in the entire study population as well as in the AHF subgroup was very reliable. Third, we were able to demonstrate that the prognostic ROC optimal cutoff of PCT to detect mortality in the entire study population and in AHF patients was 0.08 ng/mL, and hence far below the diagnostic PCT cutoff of 0.25 ng/mL established for the guidance of antibiotic therapy [9]. Importantly, the prognostic performance of PCT at this relatively low cutoff value was excellent, as patients with PCT levels > 0.08 ng/mL had a significantly higher mortality than those with PCT levels ≤ 0.08 ng/mL. Finally, we showed that the prognostic performance of PCT was fully independent of overt respiratory or other infection. Indeed, even in the AHF subgroup without any concomitant infection, mortality was significantly higher in patients with PCT levels > 0.08 ng/mL than in those with PCT levels ≤ 0.08 ng/mL.

Our study is distinct to previous trials in two major aspects.

First, the differentiation between infection and no infection was possible with a very high level of certainty, owing to the detailed diagnostic work-up and the elaborated retrospective adjudication of final diagnoses. CRP and WBC values were, for instance, normal in AHF patients without any infection, whereas they were significantly elevated in AHF patients with respiratory or other infection. This is, in fact, a pre-requisite for analyzing the prognostic role of PCT in AHF patients without respiratory or other infection. By contrast, Demissei et al. defined patients without clinical infection signs merely by temperature ≥ 38 °C or sepsis or active infection requiring intravenous antibiotic therapy at admission, and a detailed diagnostic work-up was not performed [22]. The diagnosis was also already made at admission and was not revised during hospital stay. A similar limitation was present in the study of Villanueva et al. [23]. Moreover, our study cohort comprised a relatively high percentage of AHF patients compared to the study population of the BACH trial (44.6% vs. 34.6% in the BACH trial) [24], and only 10.1% of AHF patients had respiratory or other infection. Considering the high accuracy of final diagnoses as well as the considerable amount of AHF patients without concomitant infection, our study population was thus particularly suitable to analyze the association between infection-independent, elevated PCT and increased mortality in AHF patients. For similar reasons, our study cohort was also ideal to study the diagnostic and prognostic performances of NT-proBNP. ROC statistics revealed that in our study population, NT-proBNP had excellent diagnostic performances at the established age-dependent AHF rule-in cutoffs [4,19]. Accordingly, an AUC of 0.945 (95% CI: 0.784–0.996) for patients aged < 50 years, of 0.910 (95% CI: 0.856–0.949) for patients aged between 50 and 75 years, and of 0.834 (95% CI: 0.755–0.896) for patients ages > 75 years could be evaluated. Furthermore, NT-proBNP was able to predict mortality in our study cohort. Hence, patients with NT-proBNP levels ranging above the ROC optimal cutoff of 1195 pg/mL [6] or the established age-dependent AHF rule-in cutoffs [4,19] had a higher all-cause mortality than those with values below these thresholds (log-rank *p* = 0.0182 or log-rank *p* = 0.0237, respectively). We also evaluated the diagnostic performance of PCT to detect respiratory or other infection. The diagnostic performance of PCT was noteworthy within the overall study collective (AUC = 0.774, 95% CI: 0.723–0.819) and within AHF patients (AUC = 0.801, 95% CI: 0.725–0.864). We calculated an ROC optimal cutoff value of 0.1 ng/mL for PCT in the overall study cohort and of 0.11 ng/mL in AHF patients. Applying the 0.25 ng/mL PCT cutoff established for the guidance of antibiotic therapy [9], the sensitivity decreased from 64.1% (95% CI: 47.2–78.8%) to 25.6% (95% CI: 13.0–42.1%) in the overall study cohort, and from 71.4% (95% CI: 41.9–91.6%) to 28.6% (95% CI: 8.4–58.1%) in AHF patients. However, the positive predictive value, more relevant than the sensitivity for the guidance of antibiotic therapy, increased from 46.3% (95% CI: 36.3–56.7%) to 62.4% (95% CI: 39.0–81.2%) in the overall study population, and from 40.0% (95% CI: 27.2–54.3%) to 66.7% (95% CI: 28.7–90.9%) in AHF patients. Despite the relatively small number of infections within the study population (12.5% within the overall study cohort and 10.1% within AHF patients), we were able to show that PCT could reliably detect infections. Finally, we wanted to analyze if the combination of NT-proBNP and PCT provides additional diagnostic value. We could indeed demonstrate that the diagnostic performance of NT-proBNP + PCT (AUC = 0.949, 95% CI: 0.903–0.977) was superior to that of PCT alone (AUC = 0.822, 95% CI: 0.754–0.877) for the detection of the combined diagnosis AHF and infection. However, no superiority could be shown compared to NT-proBNP alone (AUC = 0.955, 0.911–0.981). Hence, NT-proBNP added further diagnostic value (ΔAUC = 0.127) to PCT for the detection of AHF with respiratory or other infection.

Our study differs, moreover, from previous trials by the fact that we did not validate cutoffs for PCT established for the guidance of antibiotic therapy but that we tested the ability of PCT to predict all-cause mortality, regardless of concomitant infection. PCT is usually considered as a marker for bacterial load [25,26,27], which strongly correlates with the severity of infections [28,29,30,31]. Despite this, it is nevertheless primarily a marker for overall inflammation as it is produced as a response to released inflammatory mediators such as the cytokines interleukin (IL)-1β, tumor necrosis factor (TNF)-α, and IL-6 [30]. Even if these inflammatory mediators have a strong correlation with bacterial infections [32], they also have been found to be elevated in heart failure [33,34,35,36,37]. Anker et al. expounded the hypothesis that inflammation in heart failure is due to mesenteric congestion [38]. As a result, bacteria migrate from intestines to the blood stream and hence cause endotoxemia, which triggers an inflammatory response by the upregulation of cytokines. Based on that hypothesis, Niebauer et al. were the first to show that inflammatory cytokine levels increased in patients with AHF and edema [39]. They also demonstrated that PCT levels were higher in patients with AHF than in patients with compensated heart failure or without heart failure at all [39]. Further studies also supported the role of PCT as a marker of systemic inflammation by proving that it was elevated in AHF patients, even if bacterial infections were absent [11,40]. Despite the shown association of increased PCT values and AHF, the prognostic value of PCT in AHF patients without any established infection still appears to be controversial [8]. The main aim of our study was therefore to test the ability of PCT to predict all-cause mortality, irrespective of underlying respiratory or other infection. Accordingly, we were able to demonstrate that the prognostic ROC optimal PCT cutoff was 0.08 ng/mL in the entire study population and in AHF patients, and thus far below the diagnostic PCT cutoff of 0.25 ng/mL established for the guidance of antibiotic therapy [9]. Importantly, at this relatively low cutoff value, PCT was able to predict mortality in the entire study population (log-rank *p* = 0.0177) and in AHF patients (log-rank *p* = 0.0123). To exclude the influence of respiratory or other infection on PCT regarding mortality prediction, we tested the prognostic performance of PCT in AHF patients with and without respiratory or other infection. We showed that the prognostic ROC optimal PCT cutoffs were 0.08 ng/mL in AHF patients without any respiratory or other infection, and 0.22 ng/mL in AHF patients with concomitant infection. Despite a relatively low PCT cutoff level, the prognostic performance of PCT in uncomplicated AHF was, interestingly, not impeded. In AHF patients without any respiratory or other infection, mortality was indeed higher in patients with PCT levels > 0.08 ng/mL than in those with PCT levels ≤ 0.08 ng/mL (log-rank *p* = 0.0001). Similar results were obtained for AHF patients with concomitant infection at a PCT cutoff of 0.22 ng/mL (log-rank *p* = 0.0448). Furthermore, the probit regression analysis showed a significant rise of mortality probability upon PCT level increase in AHF patients with respiratory or other infection (*p* = 0.0470). Importantly, this interaction between the significant rise of mortality and the PCT level increase could also be seen in AHF patients without respiratory or other infection (*p* = 0.0001). In sum, we were able to show that PCT can predict all-cause mortality at relatively low PCT cutoff levels, irrespective of underlying respiratory or other infection. Moreover, it could predict mortality independently from NT-proBNP, as shown by the Cox regression analysis.

## 5. Conclusions

In this prospective study, which enrolled 312 patients presenting with acute dyspnea to the ED, we showed that low-level elevations of PCT below the cutoff of 0.25 ng/mL, established for the guidance of antibiotic therapy [9], are associated with a higher all-cause mortality in AHF patients, even in the absence of overt infection. These results support a recent hypothesis that PCT might be stimulated by heart failure itself or by a less relevant infection that escaped diagnosis using routine diagnostic tools [8,11,33,34,35,36,37,40]. These findings are of great practical importance because they allow an early risk stratification of dyspneic patients and support a broader use of PCT, regardless of the suspicion of a relevant respiratory or other infection.

### Limitations

Our prospective study was small, comprising only 139 patients with AHF, 14 cases with severe respiratory infections, and only 25 deaths within 120 days. Although our findings consistently support the hypothesis that prognostically relevant low-level PCT elevations may be encountered in AHF without respiratory or other infection, these observations require external validation in larger AHF cohorts.

Although we actively sought signs of bacterial infection, we cannot fully exclude that the low-level elevation of PCT might be attributed to unrecognized infection. However, this uncertainty, despite extensive work-up, does not only apply to our study but represents an issue that is probably even more relevant in clinical practice.

## Figures and Tables

**Table 1:** Characteristics of AHF patients with and without respiratory or other infection.

Parameter	AHF Patients without Respiratory or Other Infection *n* = 125	AHF Patients with Respiratory or Other Infection *n* = 14	*p*-Value
Gender (male), *n* (%)	90 of 125 (72.0%)	11 of 14 (78.6%)	0.6022
Arterial hypertension, *n* (%)	103 of 125 (82.4%)	12 of 14 (85.7%)	0.7565
Dyslipidemia, *n* (%)	73 of 125 (58.4%)	6 of 14 (42.9%)	0.2672
Diabetes mellitus, *n* (%)	49 of 125 (39.2%)	3 of 14 (21.4%)	0.1941
History of smoking, *n* (%)	83 of 125 (66.2%)	9 of 14 (66.2%)	0.8745
Obesity (BMI ≥ 30 kg/m^2^), *n* (%)	41 of 124 (33.1%)	6 of 14 (42.9%)	0.4652
Impaired systolic LV function, *n* (%)	94 of 121 (77.7%)	10 of 13 (76.9%)	0.9502
Kidney failure (GFR < 60/mL), *n* (%)	67 of 125 (53.6%)	9 of 14 (54.7%)	0.4479
Age (a), mean ± SEM	72.9 ± 1.0	74.1 ± 2.2	0.7086
NT-proBNP (ng/L), mean ± SEM	8307.4 ± 819.0	24,102.6 ± 6930.8	<0.001 *
PCT (ng/mL), mean ± SEM	0.10 ± 0.03	0.39 ± 0.23	<0.001 *
CRP (mg/L), mean ± SEM	7.7 ± 0.8	85.7 ± 18.7	<0.001 *
WBC (*n*/nL), mean ± SEM	9.6 ± 0.8	14.0 ± 1.2	<0.001 *

Abbreviations: AHF = acute heart failure; GFR = glomerular filtration rate; BMI = body mass index; LV = left ventricular; NT-proBNP = N-terminal pro brain natriuretic peptide; PCT = procalcitonin; CRP = C-reactive protein; WBC = white blood cells; SEM = standard error of the mean; * = statistically significant (*p* < 0.05).

**Table 2:** Diagnostic performances of NT-proBNP and PCT at the cutoff values.

Biomarker	Sensitivity (95% CI)	Specificity (95% CI)	Positive Predictive Value (95% CI)	Negative Predictive Value (95% CI)	Cutoff Value	AUC (95% CI)
NT-proBNP Patient’s age: <50 years, *n* = 27	100% (47.8 to 100%)	81.8% (59.7 to 94.8%)	55.5% (34.0 to 75.2%)	100%	450 ng/L (established cutoff value)	0.945 (0.784 to 0.996)
NT-proBNP Patient’s age: 50–75 years, *n* = 165	92.5% (83.4 to 97.5%)	72.5% (62.5 to 81.0%)	69.7% (62.3 to 76.2%)	93.4% (85.8 to 97.1%)	900 ng/L (established cutoff value)	0.910 (0.856 to 0.949)
NT-proBNP Patient’s age: >75 years, *n* = 120	80.6% (69.1 to 89.2%)	69.8% (55.7 to 81.7%)	77.1% (68.8 to 83.8%)	74.0% (62.9 to 82.7%)	1800 ng/L (established cutoff value)	0.834 (0.755 to 0.896)
PCT Overall study population, *n* = 312	25.6% (13.0 to 42.1%)	97.8% (95.3 to 99.2%)	62.4% (39.0 to 81.2%)	90.2% (88.4 to 91.7%)	0.25 ng/mL (established cutoff value)	0.774 (0.723 to 0.819)
PCT AHF patients, *n* = 139	28.6% (8.4 to 58.1%)	98.4% (94.3 to 99.8%)	66.7% (28.7 to 90.9%)	92.5% (89.8 to 94.5%)	0.25 ng/mL (established cutoff value)	0.801 (0.725 to 0.864)
PCT Overall study population, *n* = 312	64.1% (47.2 to 78.8%)	89.4% (85.1 to 92.8%)	46.3% (36.3 to 56.7%)	94.6% (92.0 to 96.4%)	0.10 ng/mL (ROC optimal cutoff value)	0.774 (0.723 to 0.819)
PCT AHF patients, *n* = 139	71.4% (41.9 to 91.6%)	88.0% (81.0 to 93.1%)	40.0% (27.2 to 54.3%)	96.5% (92.3 to 98.4%)	0.11 ng/mL (ROC optimal cutoff value)	0.801 (0.725 to 0.864)

Abbreviations: NT-proBNP = N-terminal pro-brain natriuretic peptide; PCT = procalcitonin; CI = confidence interval; AUC = area under the curve; ROC = receiver operating characteristic.

**Table 3:** Comparison of diagnostic performances of NT-proBNP, PCT, and NT-proBNP + PCT.

Biomarker	AUC (95% CI)	ΔAUC = AUC_NT-proBNP_–AUC_Biomarker_ (95% CI; *p*-Value)	ΔAUC = AUC_NT-proBNP + PCT_–AUC_Biomarker_ (95% CI; *p*-Value)
NT-proBNP	0.955 (0.911 to 0.981)	0	−0.006 (−0.003–0.015; *p* = 0.1615)
PCT	0.822 (0.754 to 0.877)	0.133 (0.005 to 0.262; *p* = 0.0412)	0.127 (0.003 to 0.251; *p* = 0.0440)
NT-proBNP + PCT	0.949 (0.903 to 0.977)	0.006 (0.003 to 0.015; *p* = 0.1615)	0

Abbreviations: NT-proBNP = N-terminal pro brain natriuretic peptide; PCT = procalcitonin; AHF = acute heart failure; CI = confidence interval; AUC = area under the curve.

**Table 4:** Independent prognostic performance of NT-proBNP and PCT.

Biomarker	*p*-Value
PCT retains NT-proBNP positive patients with increased mortality	0.0205
NT-proBNP retains PCT positive patients with increased mortality	0.0372

Abbreviations: NT-proBNP = N-terminal pro brain natriuretic peptide; PCT = procalcitonin.

## Data Availability

The data presented in this study are available on request from the corresponding author.
